# Supplementary material for: Dye Stabilization and Wavelength Tunability in Lasing Fibers Based on DNA
Source: Adv Opt Mater. 2020 Sep 16;8(22):2001039. doi: 10.1002/adom.202001039 (PMC7745753; doi:10.1002/adom.202001039)
Supplement: Supplementary file 1 — Supporting Information [file ADOM-8-2001039-s001.pdf]

© 2020 Wiley-VCH GmbH

ADVANCED OPTICAL MATERIALS

Supporting Information

for *Adv. Optical Mater.,* DOI: 10.1002/adom.202001039

Dye Stabilization and Wavelength Tunability in Lasing Fibers Based on DNA

Luana Persano, Adam Szukalski, Michele Gaio, Maria Moffa, Giacomo Salvadori, Lech Sznitko, Andrea Camposeo, Jaroslaw Mysliwiec, Riccardo Sapienza, Benedetta Mennucci, and Dario Pisignano**

© 2020 Wiley-VCH GmbH

Supporting Information

Dye stabilization and wavelength tunability in lasing fibers based on DNA

Luana Persano, Adam Szukalski, Michele Gaio, Maria Moffa, Giacomo Salvadori, Lech Sznitko, Andrea Camposeo, Jaroslaw Mysliwiec, Riccardo Sapienza, Benedetta Mennucci and Dario Pisignano

Dr. L. Persano, Dr. M. Moffa, Dr. A. Camposeo, Prof. D. Pisignano
NEST, Istituto Nanoscienze-CNR and Scuola Normale Superiore,
Piazza S. Silvestro 12, I-56127 Pisa, Italy
e-mail: andrea.camposeo@cnr.it
Dr. A. Szukalski, Dr. L. Sznitko, Prof. J. Mysliwiec
Faculty of Chemistry, Wroclaw University of Science and Technology,
Wybrzeze Wyspianskiego 27, 50-370 Wroclaw, Poland
Dr. M. Gaio, Prof. R. Sapienza
The Blackett Laboratory, Department of Physics,
Imperial College London, London SW7 2AZ, UK.
Mr. G. Salvadori, Prof. B. Mennucci
Department of Chemistry, University of Pisa
Via G. Moruzzi 13, I-56124 Pisa, Italy
Prof. D. Pisignano
Dipartimento di Fisica, Università di Pisa
Largo B. Pontecorvo 3, I-56127 Pisa, Italy
e-mail: dario.pisignano@unipi.it

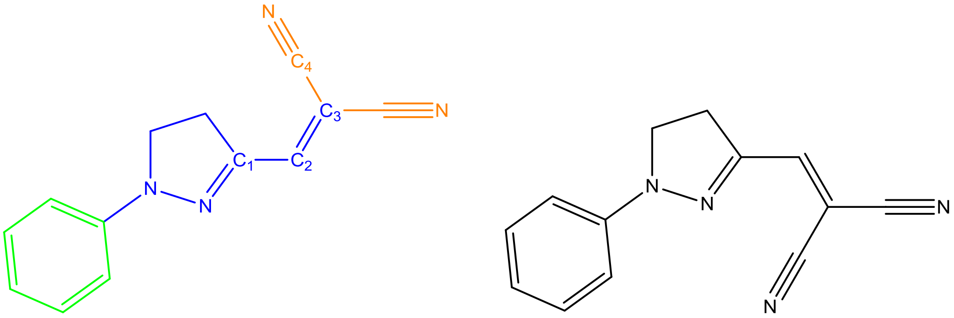

Figure S1. Molecular structure of [3-(1,1-dicyanoethenyl)-1-phenyl-4,5-dihydro-1H-pyrazole] (DCNP). Green: electron donor sub-unit, red: electron acceptor sub-unit. Blue: network of conjugated bonds that connect the two parts. The *S-trans* isomer and the *S-cis* isomers are shown in the left and in the right part of the figure, respectively.

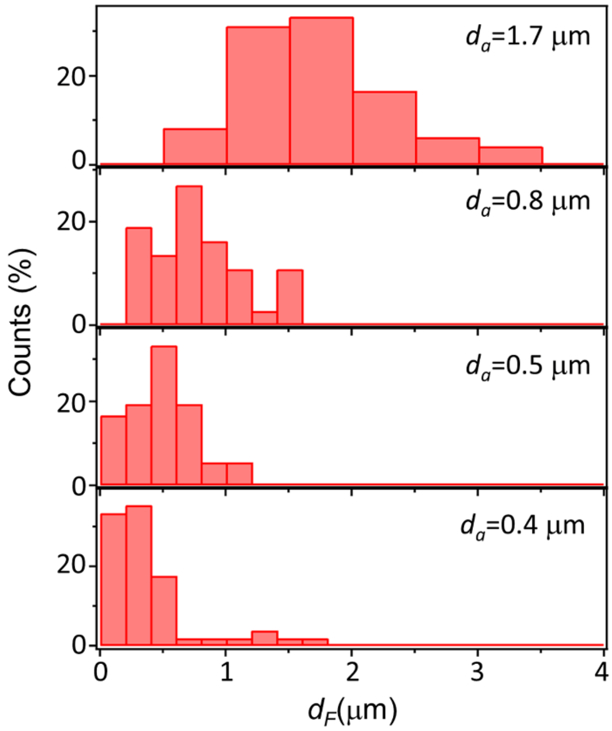

Figure S2. Distribution of the transversal size, d_F (~r_2, long cross-sectional axis) of DCNP/DNA-CTMA fibers. From top to bottom, the DNA-CTMA concentration in the solution used for electrospinning is 4%, 3.5%, 3%, 2% w/w, respectively. d_a: average value of d_F measured for the various distributions.

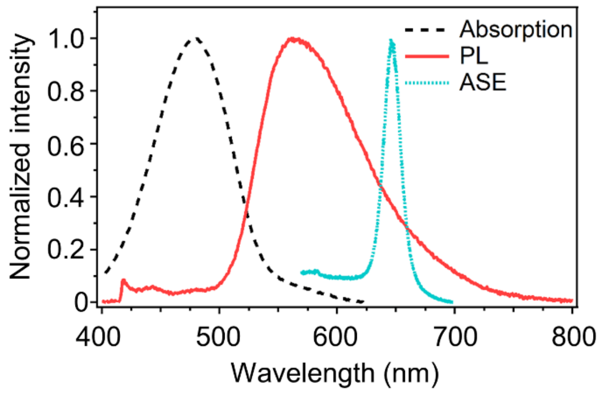

Figure S3. Absorption (black dashed line), photoluminescence (PL, red continuous line) and amplified spontaneous emission (ASE, blue dotted line) spectra of DCNP in DNA-CTMA.

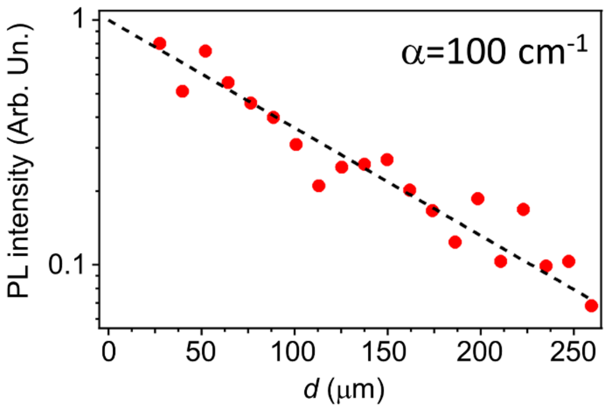

Figure S4. Decay of the light intensity (circles) guided along a DCNP/DNA-CTMA nanofiber, as a function of distance, *d*, from the excitation spot. The fiber is excited by a tightly focused laser beam. The dashed line is a fit to the data by an exponential function, $I_{PL}=I_0\times\exp(-\alpha d)$. The obtained loss coefficient, α, is about 100 cm^{-1}.

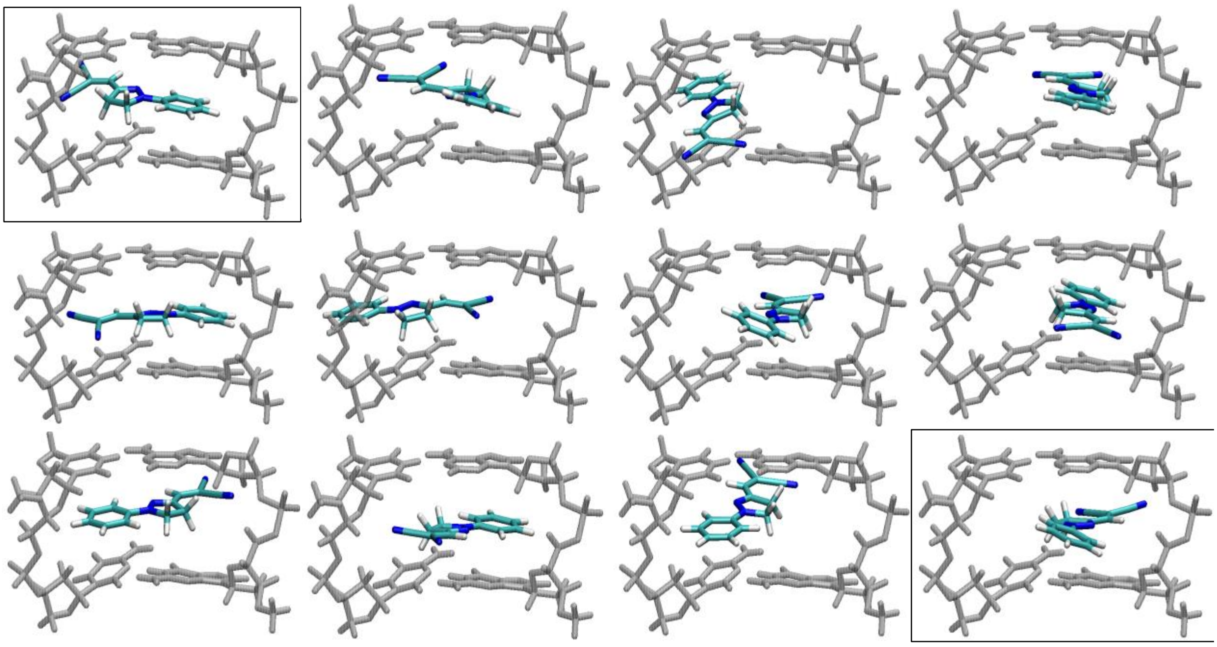

Figure S5. Representation of the 12 structures obtained by an initial screening, performed at semiempirical level (PM6), of sandwich-like and rotated intercalation modes. In grey we show the DNA model, and in color the DCNP. The two structures in the box are the two showing the lowest energy for the sandwich-like and for the rotated configuration, respectively. These two structures were finally re-optimized at DFT level (B3LYP/6-31G(d)) with the addition of empirical dispersion corrections.
